# An Adaptive Estimation Method for Heterogeneous Group Targets with Uncertain Multiplicative and Additive Noises

**DOI:** 10.3390/s26175429

**Published:** 2026-08-27

**Authors:** Zhongkai Liu, Wei Jing, Peng Wang, Wenrui Gu, Tianli Ma

**Affiliations:** 1 Nanjing Glaway Software Co., Ltd., Nanjing 211000, China; dx_dx11@163.com; 2 School of Electronic Information Engineering, Xi’an Technological University, Xi’an 710021, China; jingwei@xatu.edu.cn (W.J.); guwenrui@st.xatu.edu.cn (W.G.); matianli111@xatu.edu.cn (T.M.)

**Keywords:** heterogeneous group target, multiplicative noise, additive noise, variational Bayesian

## Abstract

**Highlights:**

**What are the main findings?**

- A unified variational Bayesian framework is proposed to jointly estimate the kinematic state, extended shape, unknown process noise covariance, and unified measurement noise covariance.
- The analytically intractable measurement likelihood is effectively approximated by a tailored hierarchical Gaussian–Gamma model, capturing the heavy-tailed characteristics induced by multiplicative noise and enabling tractable closed-form VB updates.

**What are the implications of the main findings?**

- Unlike existing VB-based filters that focus solely on additive noise, the proposed method simultaneously handles both multiplicative and additive noises with unknown and time-varying covariances, making it applicable to a broader class of practical systems such as radar tracking and cyber–physical systems.
- The proposed algorithm establishes a variational Bayesian inference framework that jointly estimates the target kinematic state and extent by closed-form analytical updates, eliminating the need for separate noise estimation modules and avoiding error propagation.

**Abstract:**

In this paper, an adaptive estimation algorithm for heterogeneous group targets considering uncertain multiplicative and additive noises is proposed. Firstly, a state-space model for heterogeneous group targets with composite multiplicative and additive noise is established. Since the coupling effect of multiplicative noise renders the marginal likelihood analytically intractable and induces heavy-tailed characteristics, a tailored hierarchical Gaussian–Gamma model is introduced for robust approximation. Second, a joint posterior probability density function incorporating the target kinematic state, extended morphology, and noise parameters is constructed. Within the variational Bayesian framework, approximate posterior distributions of these variables are derived, and fixed-point iteration is employed to compute the system state and noise statistics. Simulation results demonstrate that, under environments corrupted by unknown and time-varying multiplicative and additive noises, the proposed algorithm adaptively estimates a unified measurement noise covariance, achieving superior estimation performance compared to the random matrix model and the VB-EOT-SN method.

## 1. Introduction

Optimal filtering methods for systems contaminated by multiplicative noise have attracted increasing attention in recent years [1]. Systems corrupted by multiplicative noise are widely applied in petroleum seismic exploration [2], underwater signal processing [3], radar target tracking [4,5,6], and image processing [7]. Especially in cyber–physical systems [8,9], multiplicative noise can be regarded as malicious attacks, which may cause severe impacts on system performance. Different from additive measurement noise, the second-order and higher-order statistics of multiplicative noise are generally unknown and difficult to estimate, since they depend on the true system state. Unlike additive noise, the mean value of multiplicative noise is usually non-zero, which introduces additional challenges to state estimation [2,4,5,10].

Among adaptive filtering estimation methods for group targets, the Kalman filter serves as the optimal minimum-variance state estimator for linear systems with Gaussian process and measurement noise [11]. Benefiting from its real-time, optimal, and stable properties, it has been widely applied in communication engineering, signal processing, and aerospace engineering.

The practical performance of the Kalman filter is constrained by prior information, including noise statistics and system model parameters. Moreover, the statistical characteristics of noise are usually assumed to be known and time-invariant. Unknown prior information may degrade the estimation performance of the conventional Kalman filter. On the other hand, in many practical applications such as navigation and positioning systems [12,13,14,15], the prior knowledge of noise statistics and model parameters may be unknown, partially known, or even time-varying.

A classical solution to such problems is the adaptive Kalman filter (AKF), which can be further divided into four categories: maximum likelihood method, Bayesian method, covariance matching method, and correlation method [16]. Typical implementations of adaptive Kalman filters include the innovation-based adaptive filter [17], the interacting multiple model (IMM) filter [17,18], and the Sage–Husa adaptive filter [19].

In particular, the innovation-based adaptive filter adopts the innovation sequence and maximum likelihood criterion to estimate the noise covariance matrices. The interacting multiple model filters belong to the Bayesian framework, and other relevant methods can be regarded as its approximations. The Sage–Husa adaptive filter recursively estimates noise statistics based on the maximum a posteriori criterion and falls into the category of covariance matching methods. Nevertheless, such adaptive Kalman filters may suffer from divergence, practical application limitations, and heavy computational burden [20,21].

In recent years, variational Bayesian (VB) inference has attracted widespread attention owing to its capability of efficiently performing approximate posterior inference and estimating uncertain hidden parameters or state variables [22,23], with extensive applications in machine learning, visual tracking, signal processing, and other fields [24,25,26,27]. These existing VB methods are regarded as approximate extensions of Bayesian inference, and they are developed to estimate the additive measurement noise covariance matrix (AMNCM) by adopting appropriate conjugate prior probability density functions (PDFs) or distributions [10,28,29].

Benefiting from the strong adaptability of Bayesian and VB inference, numerous research achievements have been reported recently. In particular, a theoretical framework for VB-based filters was proposed in [30]. On this basis, various filter variants have been successively developed, including the VB-based extended Kalman filter [31], unscented Kalman filter [32], cubature information filter [33], and particle filter (PF) [34,35]. These VB-based filters generally model the additive Gaussian process and measurement noise using the heavy-tailed Student’s t-distribution, while modeling the AMNCM or additive process noise covariance matrix (APNCM) via the Wishart distribution, inverse Wishart distribution, or inverse Gamma distribution.

Nevertheless, the problem of group target tracking considering multiplicative noise within the VB inference framework remains unsolved. Since multiplicative noise can characterize a broader class of physical systems, this paper adopts VB inference to address the adaptive estimation problem of the unknown multiplicative measurement noise covariance matrix (MMNCM). In practical engineering applications, the mean and covariance of multiplicative noise are generally difficult to obtain. Accordingly, this paper proposes an adaptive estimation method for heterogeneous group targets considering uncertain multiplicative and additive noise, which is capable of adaptively estimating the unknown process noise covariance and a unified measurement noise covariance that encapsulates the effects of both multiplicative and additive noises. First, the process noise covariance, measurement noise covariance, and one-step prediction error covariance are modeled with inverse Wishart (IW) prior distributions, and the measurement likelihood is approximated by a tailored hierarchical Gaussian–Gamma model. Then, the dynamic state, extended state, and noise covariance are recursively inferred within the VB framework, and the motion state and target shape are further solved accordingly.

### Problem Formulation

Assume that there exist $n_{k}$ independent Cartesian position measurements at time k, denoted as $Z_{k}={\left\{z_{k}^{r}\right\}}_{r=1}^{n_{k}}$. For heterogeneous group targets, the following linear space model considering the coupling of multiplicative and additive noises is established:

(1) $$x_{k}=F_{k}x_{k-1}+w_{k}$$

(2) $$p(X_{k}|X_{k-1})=W(X_{k};\delta_{k},A_{k}X_{k-1}A_{k}^{T})$$

(3) $$z_{k}^{r}=m_{k}H_{k}x_{k}+v_{k}^{r},r=1,\ldots ,n_{k}$$

where $x_{k}\in {\mathbb{R}}^{n}$ is the state vector, $z_{k}^{r}\in {\mathbb{R}}^{m}$ is the measurement vector, and m and n are the measurement dimension and state dimension, respectively. $w_{k}\in {\mathbb{R}}^{n}$ and $v_{k}^{r}\in {\mathbb{R}}^{m}$ are mutually uncorrelated zero-mean Gaussian additive noises, with $w_{k}\sim N(0,Q_{k})$ and $v_{k}^{r}\sim N(0,\lambda X_{k}+R_{k})$, where λ is the model scale parameter. $R_{k}$ is the measurement noise covariance. $F_{k}\in {\mathbb{R}}^{n\times n}$ and $H_{k}\in {\mathbb{R}}^{m\times n}$ are the state transition matrix and observation matrix, respectively. $\delta_{k}>d-1$ denotes the degrees of freedom, where d is the space dimension. $A_{k}$ is an invertible matrix describing the evolution pattern, $X_{k}$ represents the extended object, and W(Y;a,C) is the density function of the Wishart distribution:

(4) $$W(Y;a,C)\propto {\left|C\right|}^{-\frac{a}{2}}{\left|Y\right|}^{\frac{a-d-1}{2}}exp[tr(-\frac{1}{2}C^{-1}Y)]$$

The key in the model lies in the multiplicative noise $m_{k}\in \mathbb{R}$ introduced in Equation (3), with a known mean ${\bar{m}}_{k}$ and an unknown covariance $\sigma_{k}$. Conditional on the vector $x_{k}$, the measurement $z_{k}^{r}$ follows an affine Gaussian distribution, with mean ${\bar{m}}_{k}H_{k}x_{k}$ and covariance $\sigma_{k}(H_{k}x_{k}){\left(H_{k}x_{k}\right)}^{T}+\lambda X_{k}+R_{k}$. However, the unconditional distribution, which involves nonlinear multiplicative coupling between the independent Gaussian random variables $m_{k}$ and $x_{k}$, no longer follows a Gaussian distribution. Instead, this unconditional distribution exhibits significant non-Gaussian heavy-tailed characteristics. Therefore, the actual statistical properties of the measurement data $z_{k}^{r}$ no longer satisfy the linear Gaussian assumptions on which standard Kalman filtering relies, leading to severe degradation in the estimation performance of conventional methods.

Furthermore, for the system described by Models (1)–(3), the Kalman filter only guarantees optimality in the linear minimum mean square error sense under the condition that $Q_{k}$, $R_{k}$ and $\sigma_{k}$ are given. However, in practical applications, the parameters $Q_{k}$, $R_{k}$ and $\sigma_{k}$ are unknown or inaccurate. Therefore, this paper proposes an adaptive estimation method for heterogeneous group targets considering uncertain multiplicative and additive noises. The proposed method can adaptively estimate the unknown process noise covariance and a unified measurement noise covariance that encapsulates the composite effects of both multiplicative and additive noises without requiring precise prior covariance information.

In the aforementioned measurement model, the measurement $z_{k}^{r}$ contains the product term of the Gaussian multiplicative noise $m_{k}$ and the system state $x_{k}$. From a statistical perspective, the probability density function of the product of two independent Gaussian random variables no longer follows a Gaussian distribution.

Theoretical studies show that the probability density function of the product of Gaussian variables has a longer “tail” than the Gaussian distribution; i.e., it exhibits significant heavy-tailed characteristics. The coupling effect of multiplicative noise renders the marginal likelihood analytically intractable and induces heavy-tailed characteristics—which, in the special zero-mean case without additive noise, can be characterized by Meijer-G distributions [36,37] only in certain simplified zero-mean, additive-noise-free cases.

**Definition** **1.** *According to the Mellin–Barnes integral representation in the complex plane, Meijer G-function is defined as*

(5) $$G_{p,q}^{m,n}(\begin{array}{c} a_{1},\ldots ,a_{p}\\ b_{1},\ldots ,b_{q} \end{array};z)=\frac{1}{2\pi i}\int_{L}\frac{\prod_{l=1}^{m}\Gamma (b_{l}-s)\prod_{l=1}^{n}\Gamma (1-a_{l}+s)}{\prod_{l=n}^{p-1}\Gamma (a_{l+1}-s)}z^{s}ds$$

*where* $a_{i}$ *and* $b_{i}$ *are real or complex parameters;* m(0⩽m⩽q) *and* n(0⩽n⩽p) *are integers; L is the integration path; i denotes the complex plane; and* Γ *denotes the Gamma function and is defined as*

(6) $$\Gamma (z)=\int_{0}^{\infty }x^{z-1}e^{-x}dx$$

Since the measurement marginal likelihood function exhibits heavy-tailed characteristics, its analytical form is extremely complicated and difficult to use directly for integral operations in Bayesian inference. If the traditional Kalman filter forces a Gaussian distribution assumption, it will ignore the outliers and heavy-tailed characteristics in the measurements, leading to a significant decline in estimation accuracy or even divergence. Therefore, it is necessary to find a distribution that can both reflect the heavy-tailed characteristics and be computationally tractable to approximate this likelihood function.

To address the analytical intractability, a tailored hierarchical Gaussian–Gamma model inspired by Student’s t-distribution is introduced as an approximate model for the measurement likelihood function.

**Definition** **2.** *Student’s t-distribution of the variable t is defined as*

(7) $$p(t;v)=\frac{\Gamma (\frac{v+1}{2})}{\sqrt{v\pi }\Gamma (\frac{v}{2})}{\left(1+\frac{t^{2}}{\nu }\right)}^{-\frac{v+1}{2}}$$

*where* Γ *is the Gamma function and* v *is the number of degrees of freedom.*

Specifically, when the degrees-of-freedom parameter v=1, Student’s t-distribution degenerates to the Cauchy distribution, whose probability density function has extremely heavy tails; when v→∞, it degenerates to the standard Gaussian distribution. This smooth transition capability from Cauchy to Gaussian enables Student’s t model to cover the measurement distribution characteristics of heterogeneous group targets under different signal-to-noise ratio (SNR) environments.

**Definition** **3.** *The Cauchy distribution of the variable* x *is defined as*

(8) $$p(x;x_{0},\gamma )=\frac{1}{\pi \gamma [1+{\left(\frac{x-x_{0}}{\gamma }\right)}^{2}]}=\frac{1}{\pi \gamma }[\frac{\gamma^{2}}{{\left(x-x_{0}\right)}^{2}+\gamma^{2}}]$$

*where* $x_{0}$ *is the location parameter and* γ *is the scale parameter.*

In this paper, the tailored hierarchical Gaussian–Gamma model is chosen to approximately characterize the measurement likelihood function, mainly based on its dual advantages in statistical robustness and the feasibility of Bayesian inference. On the one hand, Student’s t-distribution is a symmetric distribution that provides a flexible family of heavy-tailed densities through its degrees of freedom. By tuning the parameter of Student’s t-distribution, the distribution can smoothly interpolate between the Cauchy distribution and the Gaussian distribution, allowing it to effectively capture the heavy-tailed characteristics of measurement data induced by multiplicative noise coupling—a property that is particularly advantageous when the measurement likelihood deviates significantly from Gaussianity due to outliers or signal fluctuations. On the other hand, based on the normal-Gamma mixture representation theory, Student’s t-distribution can be decomposed into a conditional Gaussian process with a Gamma distribution as the prior. This hierarchical Gaussian form greatly improves the computational tractability of Bayesian inference, allowing the variational posterior distribution to be recursively updated via closed-form analytical equations. Thereby, the joint estimation of the motion state, morphology, and time-varying noise statistical characteristics of heterogeneous group targets is achieved without the need for high-dimensional numerical integration.

## 2. Proposed Algorithms

This section will be introduced from the following aspects: one-step prediction, measurement update, and algorithm derivation. The measurement update step includes the selection of the prior distribution, the selection of the likelihood function, and the selection of the covariance matrix. The algorithm derivation is based on the variational approximation of the posterior probability density function.

### 2.1. One-Step Prediction

Since the multiplicative noise does not affect the state Equation (1), the one-step prediction $p(x_{k}|Z^{k-1})$ is assumed to follow a Gaussian distribution.

(9) $$p(x_{k}|Z^{k-1},P_{k|k-1})=N(x_{k};{\hat{x}}_{k|k-1},P_{k|k-1})$$

where $Z^{k-1}={\left\{Z_{k}\right\}}_{k=0}^{k-1}$ (i.e., the accumulated sensor data up to and including time k − 1, $x_{k|k-1}$ and $P_{k|k-1}$ are the one-step predicted state vector and the corresponding one-step prediction error covariance matrix, respectively. $N\left(x;m,\Lambda \right)$ denotes a Gaussian distribution with mean vector m and covariance matrix Λ. Then

(10) $${\hat{x}}_{k|k-1}=F_{k}{\hat{x}}_{k-1|k-1}$$

(11) $$P_{k|k-1}=F_{k}P_{k-1|k-1}F_{k}^{T}+Q_{k-1}$$

where ${\hat{x}}_{k-1|k-1}$ and $P_{k-1|k-1}$ denote the state of the heterogeneous group target and the corresponding estimation error covariance matrix at time k − 1, respectively. Since $Q_{k-1}$ is unknown and linearly correlated with $P_{k|k-1}$, this paper chooses to estimate $P_{k|k-1}$ instead of $Q_{k-1}$.

**Remark** **1.** *From Equation (11),* $P_{k|k-1}$ *is a linear function of* $Q_{k-1}$*, and therefore, the estimation of* $Q_{k-1}$ *can be replaced by the estimation of* $P_{k|k-1}$.

The predicted extended density is

(12) $$p(X_{k}|Z^{k-1})=IW(X_{k};{\hat{v}}_{k|k-1},{\hat{X}}_{k|k-1})$$

where ${\hat{v}}_{k|k-1}$ and ${\hat{X}}_{k|k-1}$ are the predicted degrees-of-freedom parameter and scale matrix of the heterogeneous group target, respectively, which can be obtained via the moment matching method.

(13) $${\hat{v}}_{k|k-1}=\frac{2\delta_{k}(\lambda_{k-1}+1)(\lambda_{k-1}-1)(\lambda_{k-1}-1)}{\lambda_{k-1}^{2}(\lambda_{k-1}+\delta_{k})}+2m+4$$

(14) $${\hat{X}}_{k|k-1}=\frac{\delta_{k}}{\lambda_{k-1}}({\hat{v}}_{k|k-1}-2m-2)A_{k}{\hat{X}}_{k-1|k-1}A_{k}^{T}$$

(15) $$\lambda_{k-1}={\hat{v}}_{k-1|k-1}-2m-2$$

### 2.2. Measurement Update and Probabilistic Prior Choices

Due to the presence of multiplicative noise in the measurement equation, the distribution of the likelihood function is affected, which further influences the derivation of the measurement update step. Therefore, this paper will first discuss the selection of the likelihood function, then discuss the approximation of the noise covariance, and finally derive the proposed algorithm in the following sections.

#### 2.2.1. Characteristics and Selection of the Likelihood Function

First, consider the likelihood function $p(Z_{k}|x_{k},X_{k})$. In extended target modeling, the geometric shape $X_{k}$ of the target introduces spatial uncertainty in the origin of measurement points, which directly affects the measurement distribution. When the measurement model includes multiplicative noise and considers that measurement points originate from the shape region, the measurement model essentially involves the product of Gaussian variables and spatial random variables. The PDF of the product of independent Gaussian random variables is not necessarily a Gaussian function but exhibits heavy-tailed characteristics. Because $x_{k}$ is uncertain, marginalizing the multiplicative-state coupling yields a non-Gaussian predictive measurement distribution with heavy-tailed characteristics.

**Remark** **2.** *The convolution and the product of Gaussian probability density functions (PDFs) are also Gaussian functions, while this fact is limited to different PDFs of the same random variable.*

Second, this paper finds a method to characterize the non-Gaussian likelihood. The integral in Definition 1 is generally difficult to handle analytically, and explicit solutions for the shape parameters $\left(a_{i},b_{j}\right)$ of the Meijer G-function cannot be obtained analytically. Meanwhile, most existing special distributions are also insufficient to effectively characterize the Meijer G-function. On the other hand, an infinite mixture of Gaussian functions can approximate any distribution. Based on this property and according to Definition 2, Student’s t-distribution can be regarded as the result of an infinite Gaussian mixture, and thus can be used as one possible heavy-tailed approximation, further serving as an effective approximate representation of the Meijer G-function.

(16) $$St(x;m,\Lambda ,\nu )=\frac{\Gamma (\nu /2+1/2)}{\Gamma (\nu /2)}{\left(\frac{\Lambda }{\pi \nu }\right)}^{1/2}{\left[1+\frac{\Lambda {\left(x-m\right)}^{2}}{\nu }\right]}^{-\nu /2-1/2}$$

where m is the mean, Λ is the precision parameter, and v is the degrees of freedom. When v = 1, it reduces to the Cauchy distribution (Definition 3). In addition, as v→∞, the distribution approaches $N(x;m,\Lambda^{-1})$ with mean vector m and covariance matrix $\Lambda^{-1}$.

Third, according to the aforementioned conclusion, we use a hierarchical Gaussian–Gamma model to formulate the likelihood PDF $p(Z_{k}|x_{k},X_{k})$

(17) $$p(Z_{k}|x_{k},X_{k})=\overset{n_{k}}{\underset{r=1}{\Pi }}\int N(z_{k}^{r};m_{k}H_{k}x_{k},R_{k}^{m}/\lambda_{k}^{r}+\lambda X_{k})G(\lambda_{k}^{r};\frac{v}{2},\frac{v}{2})d\lambda_{k}^{r}$$

The covariance matrix is the sum of the scaled noise covariance term $R_{k}^{m}/\lambda_{k}^{r}$ and the structural covariance term $\lambda X_{k}$. $G(\lambda_{k}^{r};\alpha ,\beta )$ denotes the Gamma distribution of the random variable $\lambda_{k}^{r}$, where α and β are the shape parameter and rate parameter, respectively. Note that the variable $\lambda_{k}^{r}$ is distinct from the extent scale parameter λ. $\lambda_{k}^{r}$ acts only on $R_{k}^{m}$ and is independent of the target shape. This is because it is counterintuitive to scale the true physical size of the target using observation noise precision. Thus, this integral forms a tailored hierarchical model rather than a standard Student’s t-distribution. To address the non-Gaussianity of the likelihood function induced by multiplicative noise in the measurement model, as well as the problem that the coupling term $\lambda X_{k}+R_{k}$ of the extended state covariance and additive noise covariance cannot be directly computed analytically, we introduce a latent variable $y_{k}^{r}$. Then

(18) $$\begin{array}{c} p(Z_{k},Y_{k},\Lambda_{k}|x_{k},X_{k})=\prod_{r=1}^{n_{k}}p(z_{k}^{r}|y_{k}^{r},\lambda_{k}^{r})p(y_{k}^{r}|x_{k},X_{k})p(\lambda_{k}^{r})\\ =\prod_{r=1}^{n_{k}}N(z_{k}^{r}|y_{k}^{r},R_{k}^{m}/\lambda_{k}^{r})N(y_{k}^{r};m_{k}H_{k}x_{k},\lambda X_{k})G(\lambda_{k}^{r};\frac{v}{2},\frac{v}{2}) \end{array}$$

we define state variance $S_{k}≜E[x_{k}x_{k}^{T}]$. Then, m and Λ in Equation (13) are organized as

(19) $$m=m_{k}H_{k}x_{k}$$

(20) $$\Lambda =R_{k}^{m}={\bar{m}}_{k}^{2}H_{k}P_{k|k-1}H_{k}^{T}+\sigma_{k}H_{k}S_{k}H_{k}^{T}+R_{k}$$

where $Y_{k}={\left\{y_{k}^{r}\right\}}_{r=1}^{n_{k}}$, and the latent variable $y_{k}^{r}$ represents the basic noise-free measurement. Equation (18) indicates that each measurement $z_{k}^{r}$ is modeled as a noisy measurement of the noise-free point $y_{k}^{r}$ located somewhere on the extended target.

**Remark** **3.** *The tailored hierarchical Gaussian–Gamma model serves as a robust approximation rather than an exact analytical fit. Its heavy-tailed robustness is tailored to our problem via the coupled scale matrix* Λ *, while the latent precision posteriors and the unified covariance are updated during the VB iterations.*

#### 2.2.2. Choices of Covariance Matrices

Estimate the target state $x_{k}$, $X_{k}$, $P_{k|k-1}$, and the unified measurement noise covariance $R_{k}^{m}$.

In engineering practice, as shown in Equation (3), additive noise and multiplicative noise are mathematically unidentifiable from the observations alone. Therefore, we cannot estimate the AMNCM and MNNCM separately. Instead, we use an indirect method to model their combined effects as an overall measurement noise covariance matrix $p(R_{k}^{m}|Z_{k})$ and estimate them integrally using a unified covariance matrix $R_{k}^{m}$.

To ensure the consistency between the posterior probability density function and the prior probability density function, it is necessary to select appropriate conjugate prior distributions for the one-step prediction error covariance (PECM) $P_{k|k-1}$, the MMNCM $\sigma_{k}$, and the AMNCM $R_{k}$, respectively. In Bayesian analysis, the inverse Wishart distribution is usually chosen as the conjugate prior for the covariance matrix.

**Remark** **4.** *Usually, both IG distribution and IW distribution can be chosen as the conjugate prior for the covariance matrix. IG is the special case of IW distribution.*

Since $\sigma_{k}$, $P_{k|k-1}$ and $R_{k}^{m}$ are all covariance parameters in the Gaussian probability density function, to satisfy the conjugacy of the posterior and prior distributions in Bayesian inference and simplify iterative calculations, their prior probability density functions $p(P_{k|k-1}|Z^{k-1})$ and $p(R_{k}^{m}|Z^{k-1})$ are both chosen to be the IW distribution.

(21) $$p(P_{k|k-1}|Z^{k-1})=IW(P_{k|k-1};{\hat{o}}_{k|k-1},O_{k|k-1})$$

(22) $$p(R_{k}^{m}|Z^{k-1})=IW(R_{k}^{m};{\hat{u}}_{k|k-1},{\hat{U}}_{k|k-1})$$

where $IW\left(\cdot ;\mu_{k},\Sigma_{k}\right)$ denotes the IW distribution with an invertible scale matrix $\Sigma_{k}$ and degrees-of-freedom parameter $\mu_{k}$. In addition, ${\hat{o}}_{k-1|k-1}$, ${\hat{O}}_{k-1|k-1}$, ${\hat{u}}_{k-1|k-1}$ and ${\hat{U}}_{k-1|k-1}$ represent the corresponding degrees-of-freedom parameters and invertible scale matrices, respectively.

We next specify and choose the prior parameters ${\hat{o}}_{k-1|k-1}$, ${\hat{O}}_{k-1|k-1}$, ${\hat{u}}_{k-1|k-1}$ and ${\hat{U}}_{k-1|k-1}$. According to [16], the prior ${\hat{o}}_{k|k-1}$ can be chosen as

(23) $${\hat{o}}_{k|k-1}=n+\tau +1$$

where n is the dimension of states and τ≥0 is a tuning parameter. According to Equations (7) and (14), to get the prior $P_{k|k-1}$, $P_{k|k-1}$ should be set as the nominal one-step PECM ${\tilde{P}}_{k|k-1}$ as

(24) $${\tilde{P}}_{k|k-1}=\frac{{\hat{O}}_{k|k-1}}{{\hat{o}}_{k|k-1}-n-1}=F_{k}P_{k-1|k-1}F_{k}^{⊤}+{\tilde{Q}}_{k-1}$$

where ${\tilde{Q}}_{k-1}$ is the nominal APNCM. Then

(25) $${\hat{O}}_{k|k-1}=\tau {\tilde{P}}_{k|k-1}$$

For $R_{k}^{m}$ ,we choose [16]

(26) $${\hat{u}}_{k|k-1}=\rho ({\hat{u}}_{k-1|k-1}-m-1)+m+1$$

(27) $${\hat{U}}_{k|k-1}=\rho {\hat{U}}_{k-1|k-1}$$

where m is the dimension of measurements and ρ∈(0, 1] denotes a time-fluctuations forgetting factor. The initial value is

(28) $$\frac{{\hat{U}}_{0|0}}{{\hat{u}}_{0|0}-m-1}={\tilde{R}}_{0}^{m}$$

### 2.3. Iterative Filter Derivation Based on VB Inference

On the basis of the aforementioned two parts, this section will deduce the filter by using VB inference; that is, our objective is to obtain the joint posterior distribution $p(x_{k},X_{k},R_{k}^{m},Y_{k},P_{k|k-1}|Z^{k})$

(29) $$\begin{array}{cc} p(x_{k},X_{k},R_{k}^{m},Y_{k},P_{k|k-1}|Z^{k}) & \approx q(x_{k},X_{k},R_{k}^{m},Y_{k},P_{k|k-1})\\ & ≜q(x_{k})q(X_{k})q(R_{k}^{m})q(Y_{k})q(P_{k|k-1}) \end{array}$$

where q(⋅) is the approximation distribution of p(⋅).

Then, we choose to estimate $q(x_{k})q(X_{k})q(R_{k}^{m})q(Y_{k})q(P_{k|k-1})$ instead of $p(x_{k},X_{k},R_{k}^{m},Y_{k},P_{k|k-1}|Z^{k})$.

Usually, q(⋅) can be calculated by minimizing the KLD as

(30) $$\arg\min KLD[q(x_{k})q(X_{k})q(R_{k}^{m})q(Y_{k})q(P_{k|k-1})∥p(x_{k},X_{k},R_{k}^{m},Y_{k},P_{k|k-1}|Z^{k})]$$

where KLD[·] is defined in Definition 5. The solution for Equation (30) is

(31) $$\begin{array}{cc} \log q(\theta ) & =E_{\Omega^{\left(-\theta \right)}}[\log p(\Omega ,Z^{k})]+c\\ \;\;\Omega & ≜\{x_{k},X_{k},R_{k}^{m},Y_{k},P_{k|k-1}\} \end{array}$$

where θ is one element of Ω, −θ represents the complementary set of θ in Ω and c denotes the constant. Since Equation (31) cannot be solved directly, we apply the fixed-point iterative method to solve it, where $q\left(\theta \right)$ is updated after the i + 1-th iteration result $q^{i+1}\left(\theta \right)$ based on the i-th iteration.

Based on the conditional independence properties, the joint PDF $p(\Omega ,Z_{k}|Z^{k-1})$ can be obtained by

(32) $$\begin{array}{l} p(\Omega ,Z_{k}|Z^{k-1})\\ =p(Z_{k}|Y_{k},R_{k}^{m}/\lambda_{k}^{r})p(\lambda_{k}^{r})p(Y_{k}|x_{k},X_{k})\times p(x_{k},X_{k}|Z^{k-1})p(R_{k}^{m}|Z^{k-1})p(P_{k|k-1}|Z^{k-1})\\ =\prod_{r=1}^{n_{k}}N(z_{k}^{r};y_{k}^{r},R_{k}^{m}/\lambda_{k}^{r})G(\lambda_{k}^{r};\frac{v}{2},\frac{v}{2})\times \prod_{r=1}^{n_{k}}N(y_{k}^{r};m_{k}H_{k}x_{k},\lambda X_{k})\\ \times N(x_{k};x_{k|k-1},P_{k|k-1})IW(X_{k};{\hat{v}}_{k|k-1},{\hat{X}}_{k|k-1})IW(R_{k}^{m};{\hat{u}}_{k|k-1},{\hat{U}}_{k|k-1})IW(P_{k|k-1};{\hat{o}}_{k|k-1},{\hat{O}}_{k|k-1}) \end{array}$$

It then follows that $\log p(\Omega ,Z_{k}|Z^{k-1})$  is formulated as

(33) $$\begin{array}{cc} \log p(\Omega ,Z_{k} & |Z^{k-1})=\sum_{r=1}^{n_{k}}\left[(\frac{m\cdot n_{k}+v}{2}-1)\log\lambda_{k}^{r}-\frac{v}{2}\lambda_{k}^{r}\right]\\ & -\frac{1}{2}(m+{\hat{u}}_{k|k-1}+n_{k}+1)\log\left|R_{k}^{m}\right|-\sum_{r=1}^{n_{k}}\left[\frac{\lambda_{k}^{r}}{2}{\left(z_{k}^{r}-y_{k}^{r}\right)}^{⊤}{\left(R_{k}^{m}\right)}^{-1}(z_{k}^{r}-y_{k}^{r})\right]\\ & -\frac{1}{2}tr({\hat{U}}_{k|k-1}{\left(R_{k}^{m}\right)}^{-1})-\frac{1}{2}(m+{\hat{o}}_{k|k-1}+2)\left|\log P_{k|k-1}\right|\\ & -\frac{1}{2}{\left(x_{k}-{\hat{x}}_{k|k-1}\right)}^{⊤}P_{k|k-1}^{-1}(x_{k}-{\hat{x}}_{k|k-1})-\frac{1}{2}tr({\hat{O}}_{k|k-1}P_{k|k-1}^{-1})\\ & -\frac{1}{2}({\hat{v}}_{k|k-1}+m+1)\log\left|X_{k}\right|-\sum_{r=1}^{n_{k}}\left[\frac{1}{2}{\left(y_{k}^{r}-m_{k}H_{k}x_{k}\right)}^{T}{\left(\lambda X_{k}\right)}^{-1}(y_{k}^{r}-m_{k}H_{k}x_{k})\right]\\ & -\frac{1}{2}tr({\hat{X}}_{k|k-1}X_{k}^{-1})-\frac{n_{k}}{2}\log\left|X_{k}\right|+c \end{array}$$

By using $\theta =\lambda_{k}^{r}$ and substituting Equation (33) into Equation (31), we obtain

(34) $$\log q^{i+1}(\lambda_{k}^{r})=(\frac{m+v}{2}-1)\log\lambda_{k}^{r}-\frac{1}{2}\{v+\mathrm{tr}(\sum_{r=1}^{n_{k}}E^{i}[(z_{k}^{r}-y_{k}^{r}){\left(z_{k}^{r}-y_{k}^{r}\right)}^{T}{\left(R_{k}^{m}\right)}^{-1}])\}\lambda_{k}^{r}+c$$

The right-hand side of Equation (34) has the logarithmic form of a Gamma distribution. Taking the exponential of both sides of Equation (34) and normalizing, we obtain

(35) $$q^{i+1}(\lambda_{k}^{r})=G(\lambda_{k}^{r};\gamma_{k}^{i+1},\chi_{k}^{i+1})$$

where

(36) $$\gamma_{k}^{i+1}=\frac{1}{2}\left(m+v\right)$$

(37) $$\chi_{k,r}^{i+1}=\frac{1}{2}\{\mathrm{tr}(E[(z_{k}^{r}-y_{k}^{r}){\left(z_{k}^{r}-y_{k}^{r}\right)}^{T}{\left(R_{k}^{m}\right)}^{-1}])+v\}$$

(38) $$\sum_{r=1}^{n_{k}}E^{i}[(z_{k}^{r}-y_{k}^{r}){\left(z_{k}^{r}-y_{k}^{r}\right)}^{T}]=\sum_{r=1}^{n_{k}}\left[(z_{k}^{r}-y_{k}^{r,i}){\left(z_{k}^{r}-y_{k}^{r,i}\right)}^{T}+\Sigma_{k}^{y,i}\right]$$

(39) $$E^{i+1}[{\left(R_{k}^{m}\right)}^{-1}]={\hat{u}}_{k}^{i+1}{\left({\hat{U}}_{k}^{i+1}\right)}^{-1}$$

Letting $\theta =P_{k|k-1}$ and employing Equation (33) in Equation (31), we obtain

(40) $$\begin{array}{ll} \log q^{i+1}(P_{k|k-1})= & -\frac{1}{2}({\hat{o}}_{k|k-1}+m+2)\log\left|P_{k|k-1}\right|\\ & -\frac{1}{2}tr\{E{\left[(x_{k}-{\hat{x}}_{k|k-1})\right]}^{x_{k}-{\hat{x}}_{k|k-1})]T}+{\hat{O}}_{k|k-1}\}P_{k|k-1}^{-1}+c \end{array}$$

The right-hand side of Equation (40) has the logarithmic form of an inverse Wishart distribution. Taking the exponential of both sides of Equation (40) and normalizing, we obtain

(41) $$q^{i+1}(P_{k|k-1})=IW(P_{k|k-1};{\hat{o}}_{k}^{i+1},{\hat{O}}_{k}^{i+1})$$

where

(42) $${\hat{o}}_{k}^{i+1}={\hat{o}}_{k|k-1}+1$$

(43) $$\begin{array}{cc} {\hat{O}}_{k}^{i+1} & =E^{i}[(x_{k}-{\hat{x}}_{k|k-1})\left(x_{k}-{\hat{x}}_{k|k-1}\right)T]+{\hat{O}}_{k|k-1}\\ & =P_{k|k}^{i}+({\hat{x}}_{k|k}^{i}-{\hat{x}}_{k|k-1})\left({\hat{x}}_{k|k}^{i}-{\hat{x}}_{k|k-1}\right)T+{\hat{O}}_{k|k-1} \end{array}$$

Letting $\theta =R_{k}^{m}$ and using Equation (33) in Equation (31), we obtain

(44) $$\begin{array}{cc} \log q^{i+1}(R_{k}^{m})= & -\frac{1}{2}({\hat{u}}_{k|k-1}+m+n_{k}+1)\log\left|R_{k}^{m}\right|\\ & -\frac{1}{2}tr\{\sum_{r=1}^{n_{k}}\left\{[E^{i}(z_{k}^{r}-y_{k}^{r}){\left(z_{k}^{r}-y_{k}^{r}\right)}^{T}+\Sigma_{k}^{y,i}]E^{i+1}[\lambda_{k}^{r}]\right\}+{\hat{U}}_{k|k-1}\}{\left(R_{k}^{m}\right)}^{-1}+c \end{array}$$

The right-hand side of Equation (44) has the logarithmic form of an inverse Wishart distribution. Taking the exponential of both sides of Equation (44) and normalizing, we obtain

(45) $$q^{i+1}(R_{k}^{m})=IW(R_{k}^{m};{\hat{u}}_{k}^{i+1},{\hat{U}}_{k}^{i+1})$$

where

(46) $${\hat{u}}_{k}^{i+1}={\hat{u}}_{k|k+1}+n_{k}$$

(47) $${\hat{U}}_{k|k-1}=\sum_{r=1}^{n_{k}}\left\{[E^{i}(z_{k}^{r}-y_{k}^{r}){\left(z_{k}^{r}-y_{k}^{r}\right)}^{T}+\Sigma_{k}^{y,i}]E^{i+1}[\lambda_{k}^{r}]\right\}+{\hat{U}}_{k|k-1}$$

(48) $$E^{i+1}[\lambda_{k}^{r}]=\frac{\gamma_{k}^{i+1}}{\chi_{k,r}^{i+1}}$$

Letting $\theta =x_{k}$ and using Equation (33) in Equation (31), we obtain

(49) $$\begin{array}{cc} \log q^{i+1}(x_{k})= & -\frac{1}{2}{\left(x_{k}-{\hat{x}}_{k|k-1}\right)}^{T}E^{i+1}[P_{k|k-1}^{-1}](x_{k}-{\hat{x}}_{k|k-1})\\ & -\frac{1}{2}\sum_{r=1}^{n_{k}}{\left(E^{i}[y_{k}^{r}]-{\bar{m}}_{k}H_{k}x_{k}\right)}^{T}E^{i+1}[{\left(\lambda X_{k}\right)}^{-1}](E^{i}[y_{k}^{r}]-{\bar{m}}_{k}H_{k}x_{k})+c\\ & =\log N(x_{k};{\hat{x}}_{k|k-1},{\left\{E^{i+1}[P_{k|k-1}^{-1}]\right\}}^{-1})+\log N({\bar{y}}_{k};{\bar{m}}_{k}H_{k}x_{k},\frac{{\left\{E[{\left(\lambda X_{k}\right)}^{-1}]\right\}}^{-1}}{n_{k}})+c \end{array}$$

where $E^{i+1}[P_{k|k-1}^{-1}]=({\hat{o}}_{k}^{i+1}-n-1){\left({\hat{O}}_{k}^{i+1}\right)}^{-1}$, ${\bar{y}}_{k}=\frac{1}{n_{k}}\sum_{r=1}^{n_{k}}{\bar{y}}_{k}^{r}$, ${\bar{y}}_{k}^{r}≜E^{i}(y_{k}^{r})={\hat{y}}_{k}^{r,i}$ and $E[{\left(\lambda X_{k}\right)}^{-1}]≜E^{i}[{\left(\lambda X_{k}\right)}^{-1}]=({\hat{\nu }}_{k|k}^{i}-m-1){\left(\lambda {\hat{X}}_{k}^{i}\right)}^{-1}$. Taking the exponential of both sides of Equation (49), normalizing, and applying the Gaussian product formula, we obtain

(50) $$q_{x}^{i+1}(x_{k})=N(x_{k};{\hat{x}}_{k|k}^{i+1},P_{k|k}^{i+1})$$

where

(51) $$x_{k|k}^{i+1}={\hat{x}}_{k|k-1}+K_{k}^{i+1}({\bar{y}}_{k}-{\bar{m}}_{k}H_{k}{\hat{x}}_{k|k-1})$$

(52) $$P_{k|k}^{i+1}={\tilde{P}}_{k|k-1}^{i+1}-K_{k}^{i+1}H_{k}{\tilde{P}}_{k|k-1}^{i+1}$$

(53) $$K_{k}^{i+1}={\bar{m}}_{k}^{2}{\tilde{P}}_{k|k-1}H_{k}^{T}(\frac{\lambda {\hat{X}}_{k|k}^{i}}{n_{k}({\hat{v}}_{k|k}^{i}-m-1)}+{\bar{m}}_{k}^{2}H_{k}{\tilde{P}}_{k|k-1}H_{k}^{T}{)}^{-1}$$

Letting $\theta =X_{k}$ and using Equation (33) in Equation (31), we obtain

(54) $$\begin{array}{cc} \log q^{i+1}(X_{k})= & -\frac{1}{2}tr\{\sum_{r=1}^{n_{k}}E^{i+1}[(y_{k}^{r}-m_{k}H_{k}x_{k}){\left(y_{k}^{r}-m_{k}H_{k}x_{k}\right)}^{T}]{\left(\lambda X_{k}\right)}^{-1}\}\\ & -\frac{1}{2}({\hat{v}}_{k|k-1}+n_{k}+m+1)\log\left|X_{k}\right|-\frac{1}{2}tr({\hat{X}}_{k|k-1}X_{k}^{-1})\\ & =-\frac{1}{2}({\hat{v}}_{k|k-1}+n_{k}+m+1)\log\left|X_{k}\right|\\ & -\frac{1}{2}tr\{\frac{1}{\lambda }\sum_{r=1}^{n_{k}}E^{i+1}[(y_{k}^{r}-m_{k}H_{k}x_{k}{\left(y_{k}^{r}-m_{k}H_{k}x_{k}\right)}^{T}]+{\hat{X}}_{k|k-1}\}X_{k}^{-1}+c \end{array}$$

The right-hand side of Equation (54) has the logarithmic form of an inverse Wishart distribution. Taking the exponential of both sides of Equation (54) and normalizing, we obtain

(55) $$q^{i+1}(X_{k})=IW(X_{k};{\hat{v}}_{k|k}^{i+1},{\hat{X}}_{k|k}^{i+1})$$

where

(56) $${\hat{v}}_{k|k}^{i+1}={\hat{v}}_{k|k-1}+n_{k}$$

(57) $$\begin{array}{cc} {\hat{X}}_{k|k}^{i+1} & ={\hat{X}}_{k|k-1}+\frac{1}{\lambda }\sum_{r=1}^{n_{k}}E[(y_{k}^{r}-m_{k}H_{k}x_{k}){(y_{k}^{r}}^{-m_{k}H_{k}x_{k})T}]\\ & ={\hat{X}}_{k|k-1}+\frac{1}{\lambda }\sum_{r=1}^{n_{k}}[(y_{k}^{r,i}-{\bar{m}}_{k}H_{k}{\hat{x}}_{k|k}^{i})(y_{k}^{r,i}-{\bar{m}}_{k}H_{k}{\hat{x}}_{k|k}^{i})T+{\bar{m}}_{k}^{2}H_{k}P_{k|k}^{i}H_{k}^{T}+\Sigma_{k}^{y,i}] \end{array}$$

Letting $\theta =Y_{k}$ and using Equation (33) in Equation (31), we obtain

(58) $$\begin{array}{cc} \log q^{i+1}(Y_{k})= & -\sum_{k=1}^{n_{k}}\frac{1}{2}E[\lambda_{k}^{r}]\times tr\{E[(z_{k}^{r}-y_{k}^{r})(z_{k}^{r}-y_{k}^{r})]TE[(R_{k}^{m})-1]\}\\ & -\sum_{r=1}^{n_{k}}\frac{1}{2}tr\{E[(y_{k}^{r}-m_{k}H_{k}x_{k})(y_{k}^{r}-m_{k}H_{k}x_{k})T]E[(\lambda X_{k})-1]\}\\ & =\sum_{r=1}^{n_{k}}\log N(z_{k}^{r};y_{k}^{r},\frac{{\left\{E^{i+1}[{\left(R_{k}^{m}\right)}^{-1}]\right\}}^{-1}}{E^{i+1}[\lambda_{k}^{r}]})\\ & +\log N(y_{k}^{r};{\bar{m}}_{k}H_{k}x_{k},\{E^{i+1}[(\lambda X_{k})-1]\}-1)+c \end{array}$$

Taking the exponential of both sides of Equation (58), normalizing, and applying the Gaussian product formula, we obtain

(59) $$q^{i+1}(Y_{k})=\prod_{r=1}^{n_{k}}N(y_{k}^{r};{\hat{y}}_{k}^{r,i+1},\Sigma_{k}^{y,i+1})$$

where

(60) $${\hat{y}}_{k}^{r,i+1}={\bar{m}}_{k}H_{k}{\hat{x}}_{k|k}^{i}+K_{k}^{y}(z_{k}^{r}-{\bar{m}}_{k}H_{k}{\hat{x}}_{k|k}^{i})$$

(61) $$\Sigma_{k}^{y,i+1}=\frac{\lambda {\hat{X}}_{k|k}^{i+1}}{{\hat{v}}_{k|k}^{i+1}-m-1}-\frac{\lambda {\hat{X}}_{k|k}^{i+1}}{{\hat{v}}_{k|k}^{i+1}-m-1}{\left(K_{k}^{y}\right)}^{T}$$

(62) $$K_{k}^{y}=\frac{\lambda {\hat{X}}_{k|k}^{i}}{{\hat{v}}_{k|k}^{i}-m-1}\times (\frac{{\hat{U}}_{k}^{i}\cdot \chi_{k}^{i}}{({\hat{u}}_{k}^{i+1}-m-1)\gamma_{k}^{i}}+\frac{\lambda {\hat{X}}_{k|k}^{i+1}}{{\hat{v}}_{k|k}^{i}-m-1}{)}^{-1}$$

The computational complexity of the proposed algorithm per time step can be decomposed as follows: the prediction step requires matrix operations of order $O(n^{3})$; the measurement update, which involves computing the Kalman gain and innovation covariance, incurs a cost of $O(m^{3}+n^{3})$; and each VB fixed-point iteration, which sequentially updates the auxiliary variable, the extended morphology, the kinematic state, and the three covariance matrices, is dominated by processing all $N_{k}$ measurements and matrix inversions, yielding a per-iteration complexity of $O(N_{k}m^{2}+n^{3}+m^{3})$. Consequently, the total computational complexity per time step is $O(n^{3}+m^{3}+N_{\max}(N_{k}m^{2}+n^{3}+m^{3}))$. In contrast, the RMM algorithm performs only a single prediction-update cycle without iteration, resulting in a complexity of $O(N_{k}m^{2}+n^{3}+m^{3})$, while the VB-EOT-SN algorithm, which estimates only the AMNCM, has a complexity of $O(N_{\max}(N_{k}m^{2}+n^{3}+m^{3}))$ with a smaller constant factor. Algorithm 1 summarizes the proposed method.
**Algorithm 1.** The Proposed Adaptive Estimation MethodInput: Measurement $Z_{k}$, prior state ${\hat{x}}_{k-1|k-1}$, covariances $P_{k-1|k-1}$, tuning parameters ρ,τ, known multiplicative noise mean ${\bar{m}}_{k}$, max iterations $N_{\max}=10$, tolerance $\varepsilon =10^{-4}$ Output: Estimated state ${\hat{x}}_{k|k}$, extended shape ${\hat{X}}_{k|k}$, unified noise covariance ${\hat{R}}_{k}^{m}$1. Initialization: Calculate one-step predictions using Equations (10)–(15). Initialize variational parameters 2. For i=0 to $N_{\max}-1$ do: 3. Update latent variable expectations $y_{k}^{r,i+1}$ and precision $\lambda_{k}^{r,i+1}$ using Equations (35)–(39) 4. Update the unified measurement noise covariance ${\hat{R}}_{k}^{m,i+1}$ using Equations (45)–(47) 5. Update the state ${\hat{x}}_{k|k}^{i+1}$ and prediction error covariance $P_{k|k-1}^{i+1}$ using Equations (41)–(43) and (50)–(53) 6. Update the extended morphology ${\hat{X}}_{k|k}^{i+1}$ using Equations (55)–(57) 7. Check convergence: if $\left\|{\hat{x}}_{k|k}^{i+1}-{\hat{x}}_{k|k}^{i}\right\|<\varepsilon$, break 8. End For Note: No theoretical guarantee of monotonic Evidence Lower Bound increase or convergence to a local optimum is claimed due to the approximations involved. Furthermore, the updated scale matrices preserve positive-definiteness provided that the initial matrices are symmetric positive-definite, and all relevant inverse Wishart degrees of freedom satisfy the existence conditions for inverse moments, i.e., ${\hat{u}}_{k}^{i+1}>m+1$, ${\hat{o}}_{k}^{i+1}>n+1$, and ${\hat{v}}_{k|k}^{i+1}>m+1$.

## 3. Performance Analysis

**Figure 1:**
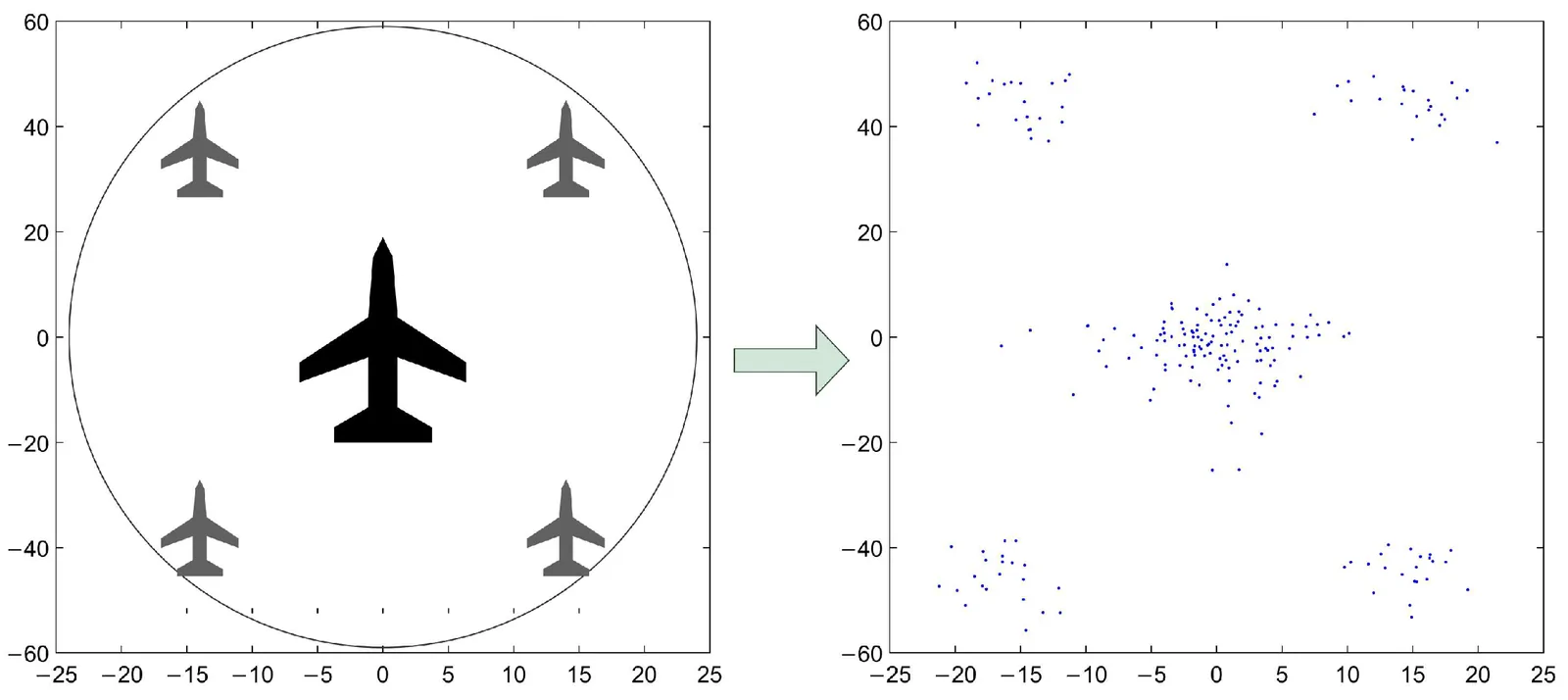
Target formation and measurement distribution.

To verify the effectiveness of the proposed algorithm, under the condition that the covariances of both additive and multiplicative noise are unknown, we conduct a comparative evaluation of extended target tracking performance using the random matrix model and the VB-EOT-SN method, respectively. The spatial distribution structure of the heterogeneous group targets is illustrated in Figure 1.

In the two-dimensional plane, the target moves with constant velocity, and its dynamic model is given by Equation (1). $x_{k}$ is the center state of the heterogeneous group target, defined as $x_{k}={\left[x_{k},y_{k},{\dot{x}}_{k},{\dot{y}}_{k}\right]}^{T}$, where $x_{k},y_{k},{\dot{x}}_{k},{\dot{y}}_{k}$ represent the position and velocity vectors of the heterogeneous group target in the X and Y directions at time k, respectively.

The group consists of five sub-targets, where the measurement number for each follows a Poisson distribution with parameter $\lambda_{i}$. Their initial spatial offsets and Poisson means are summarized in Table 1.

Assume that the initial extended state $X_{k}$ of the heterogeneous target is an ellipse with diameters of 60 m and 20 m. The measurement equation is given by Equation (3). The true additive process noise covariance and additive observation noise covariance are both slowly time-varying, which are given by the following equations:

(63) $$Q_{k}=[6.5+0.5\cos(\frac{\pi k}{\Delta t})]q\left[\begin{array}{cc} \frac{T^{3}}{3}I_{2} & \frac{T^{2}}{2}I_{2}\\ \frac{T^{2}}{2}I_{2} & TI_{2} \end{array}\right]$$

(64) $$R_{k}=[0.1+0.05\cos(\frac{\pi k}{\Delta t})]r\left[\begin{array}{cc} 1 & 0.5\\ 0.5 & 1 \end{array}\right]$$

where Δt=10 s denotes the time step, $q=1{\;m}^{2}/s^{3}$ and $r=100{\;m}^{2}$, and $I_{2}$ is the 2 × 2 identity matrix. The multiplicative noise is set as a Gaussian process with mean ${\bar{m}}_{k}=1.5$ and variance $\sigma_{k}=[0.1+0.05\cos(\frac{\pi k}{\Delta t})]r$.

In addition, the nominal APNCM and MNCM are set to ${\tilde{Q}}_{k}=\alpha I_{4}$ and ${\tilde{R}}_{0}^{M}=\beta I_{2}$, respectively, where $I_{4}$ is the 4 × 4 identity matrix. ρ=1−exp(−4), τ=100, α=1 and β=100. The three methods are provided with the same initial states, initial covariances, and prior nominal noise statistics at t=0, and run for the same number of time steps.

**Figure 2:**
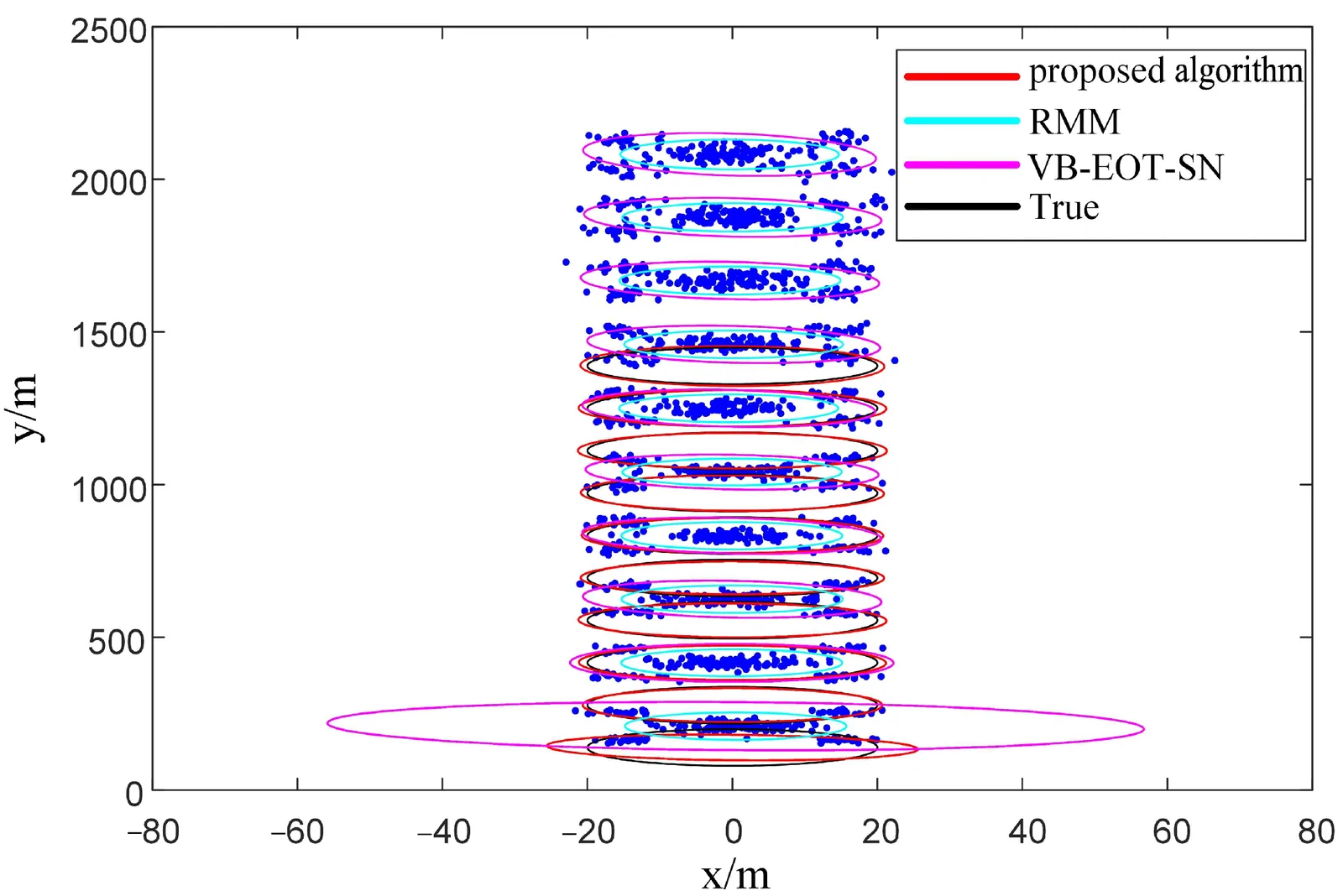
Performance comparison of three algorithms.

Figure 2 shows the tracking results of heterogeneous group targets obtained by the three algorithms in a single experiment. It can be seen that the proposed algorithm can accurately estimate the shape and state of heterogeneous group targets under the condition that the covariances of both additive and multiplicative noise are unknown, thus exhibiting better performance.

**Figure 3:**
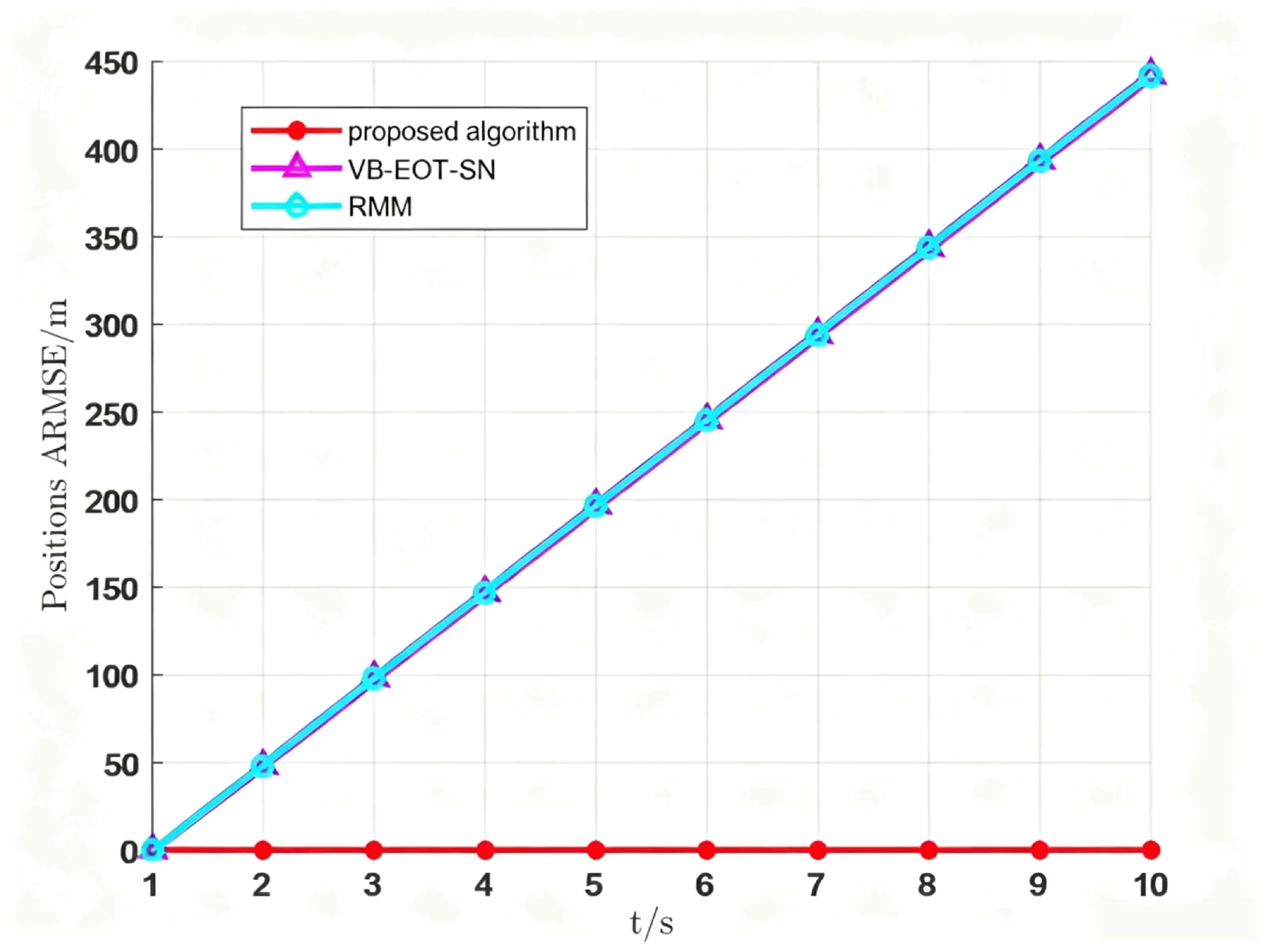
Position ARMSE comparison of three algorithms.

**Figure 4:**
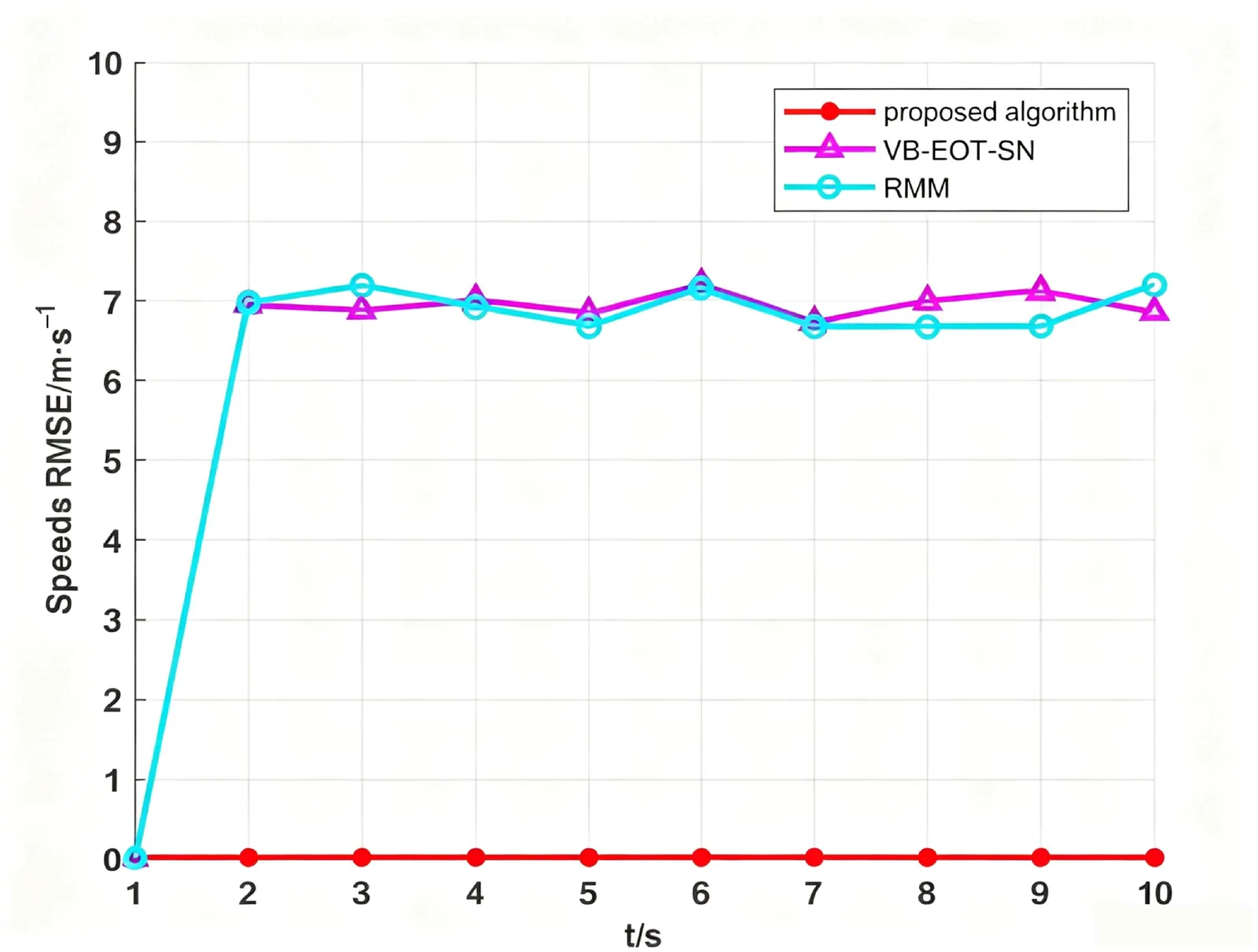
Velocity ARMSE comparison of three algorithms.

To verify the effectiveness of the proposed algorithm in the state estimation of heterogeneous group targets, the root mean square error (RMSE) of position and velocity is selected as the performance metric. The averaged metrics reported in this study are computed by averaging the specific error values across all 100 Monte Carlo simulation trials at each time step. Figure 3 presents the comparison of the average root mean square error (ARMSE) in position for the three algorithms. It can be observed that the errors of the baselines rise rapidly over time, as they fail to handle the scaling effect caused by the non-zero multiplicative noise mean. In contrast, our algorithm compensates for this and achieves a 0.07 m error. Figure 4 shows the comparison of the velocity ARMSE for the three algorithms. The errors of the RMM and VB-EOT-SN algorithms are still higher than those of the proposed algorithm. The experimental results demonstrate that the proposed algorithm exhibits excellent performance in both positioning accuracy and velocity estimation across 100 Monte Carlo simulation runs, with higher accuracy and robustness.

Table 2 presents the position and velocity ARMSE comparison of the evaluated algorithms. An Oracle RMM baseline supplied with the multiplicative mean ${\bar{m}}_{k}=1.5$ is included to eliminate the scaling mismatch. All results are reported in the mean ± SD format over 100 Monte Carlo runs to ensure statistical reliability. The experimental data show that the proposed algorithm outperforms all baselines in both position and velocity estimation. While the Oracle RMM avoids divergence, the position ARMSE of the proposed algorithm is only 0.07 m with minimal SD, which is much lower than the 0.35 m of the Oracle RMM and those of the RMM and VB-EOT-SN algorithms. In terms of velocity estimation, the ARMSE of the proposed algorithm is also superior to the 2.85 m/s of the Oracle RMM, the 5.96 m/s of the RMM algorithm, and the 5.95 m/s of the VB-EOT-SN algorithm.

To evaluate the overall fitting accuracy of the heterogeneous group target contour, the Modified Hausdorff Distance (MHD) and Intersection over Union (IoU) are adopted to verify the effectiveness of the algorithm. A smaller AMHD value indicates that the estimated shape is closer to the real target boundary. The corresponding calculation formula is given as follows:

(65) $$\begin{array}{l} d_{H}(J(x_{k}),\hat{J}({\hat{x}}_{k}))\\ =max\left\{d(J(x_{k}),\hat{J}({\hat{x}}_{k})),d\left(\hat{J}({\hat{x}}_{k}),J(x_{k})\right)\right\} \end{array}$$

where

(66) $$d(\hat{J}({\hat{x}}_{k}),J(x_{k}))=\frac{1}{\left|\hat{J}({\hat{x}}_{k})\right|}\sum_{{\hat{q}}_{k}\in \hat{J}({\hat{x}}_{k})}\left\{d_{E}({\hat{q}}_{k},J(x_{k}))\right\}$$

where $d_{E}(q_{k},J_{\hat{A}}({\hat{x}}_{k}))$ denotes the standard Euclidean distance between $q_{k}$ and ${\hat{q}}_{k}$ in $\hat{J}({\hat{x}}_{k})$, where $q_{k}$ and ${\hat{q}}_{k}$ are any points in the point sets $J(x_{k})$ and $\hat{J}({\hat{x}}_{k})$, respectively. $J(x_{k})$ and $\hat{J}({\hat{x}}_{k})$ represent the discrete estimated shape contour point set and the real contour point set obtained by uniform angular sampling at {2πi/n,i=1,…,n}, where n is the number of sampling points. $\left|\hat{J}({\hat{x}}_{k})\right|$ is the number of points in the set $\hat{J}({\hat{x}}_{k})$.

The IoU directly reflects the geometric accuracy of shape matching by calculating the ratio of the intersection area to the union area between the estimated shape and the real shape:

(67) $$IoU=\frac{S_{e}\cap S_{t}}{S_{e}\cup S_{t}}$$

where $S_{e}$ and $S_{t}$ denote the estimated ellipse area and the real ellipse area, respectively.

**Figure 5:**
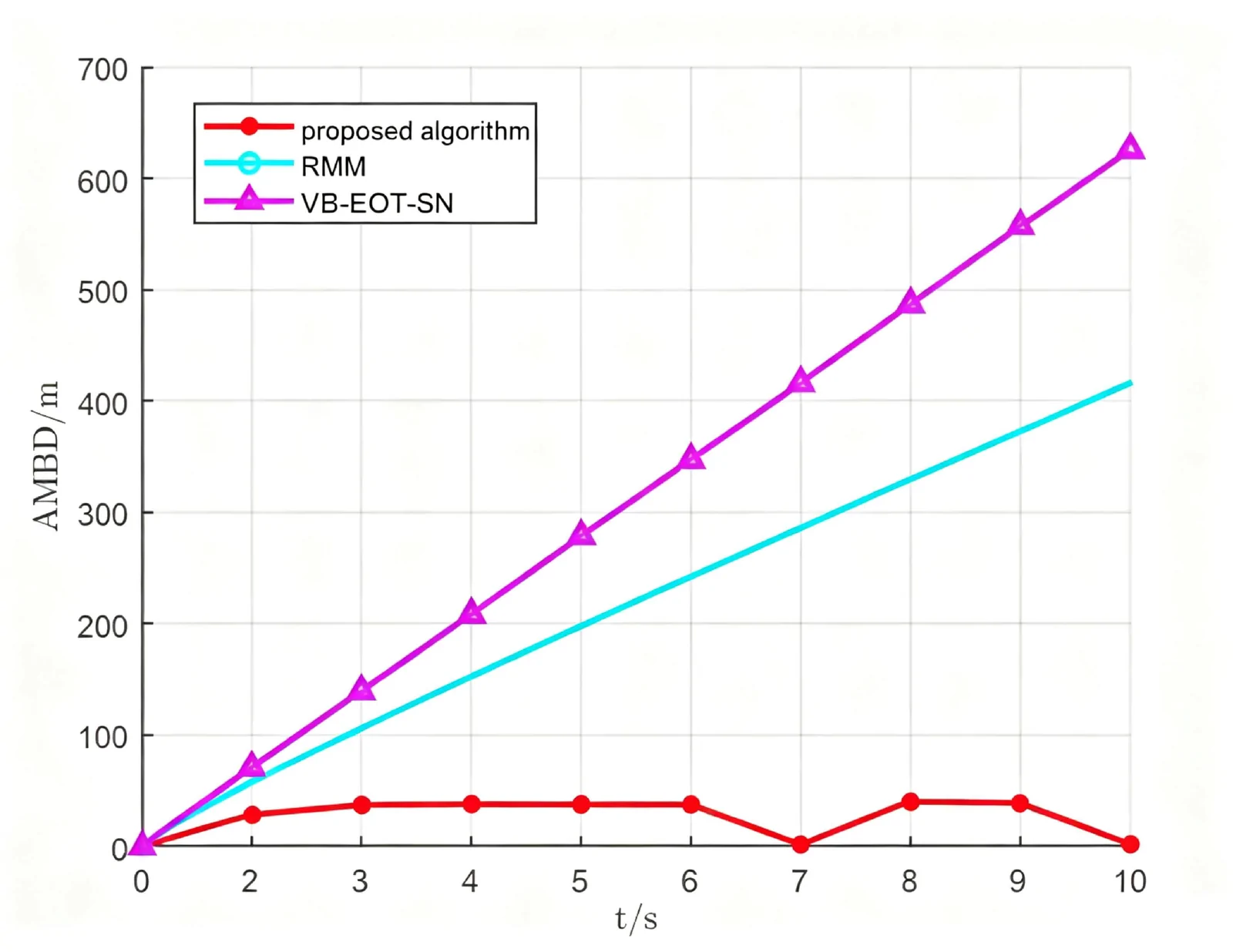
MHD comparison of three algorithms.

**Figure 6:**
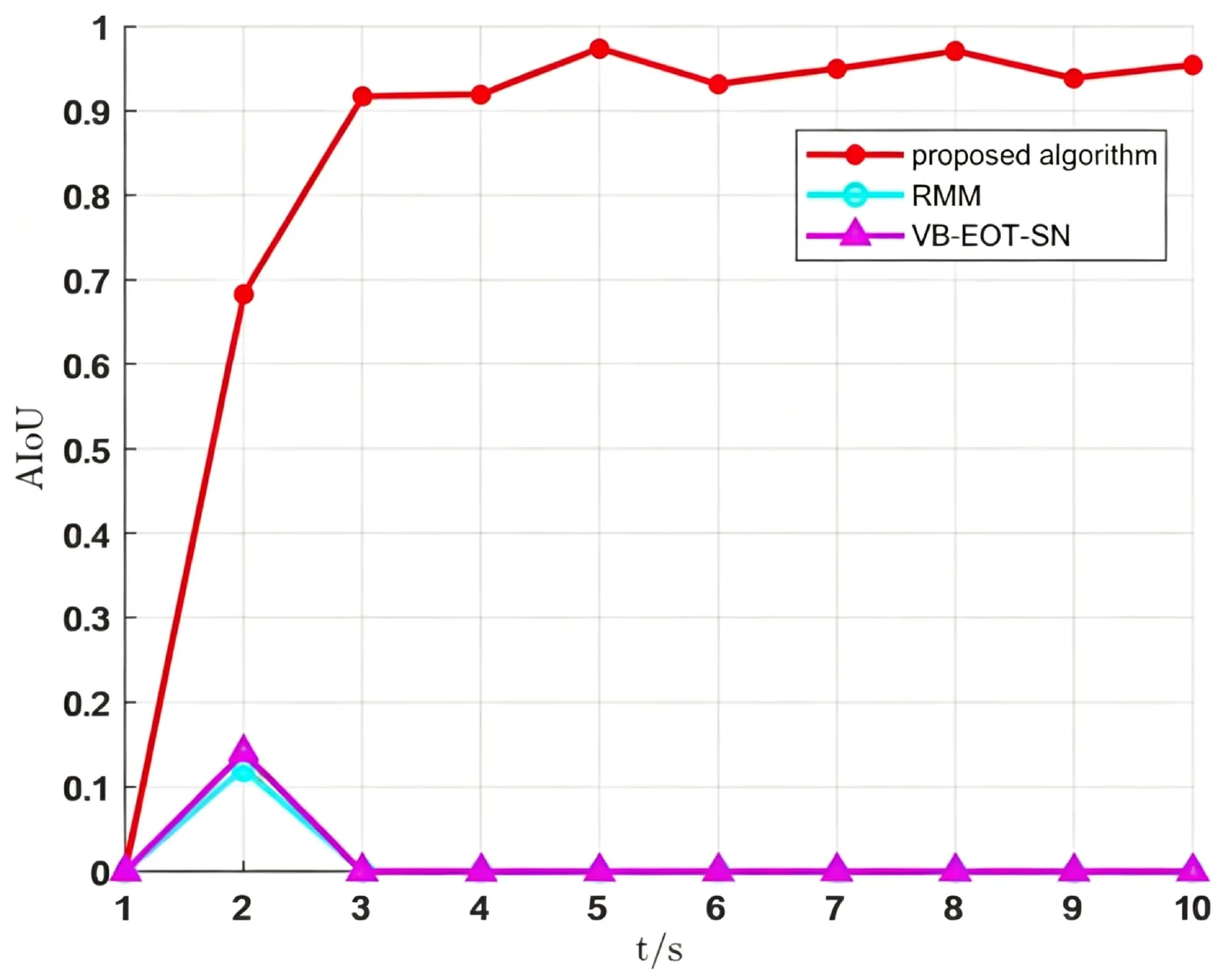
AIoU comparison of three algorithms.

Figure 5 and Figure 6 show the AMHD and AIoU comparisons of the three algorithms, respectively. It can be seen from the figures that the proposed algorithm achieves the smallest MHD and the largest IoU.

Table 3 presents the comparison of AMHD and AIoU. The results show that the AMHD of the proposed algorithm is only 26.01 m, which is lower than the 48.35 m of the Oracle RMM, 312.67 m of the RMM algorithm, and 311.78 m of the VB-EOT-SN algorithm. Meanwhile, the AIoU value of the proposed algorithm reaches 0.87, outperforming the 0.45 of the Oracle RMM and indicating that the estimated target contour has a high degree of overlap with the real shape, and thus achieves excellent shape matching performance.

**Figure 7:**
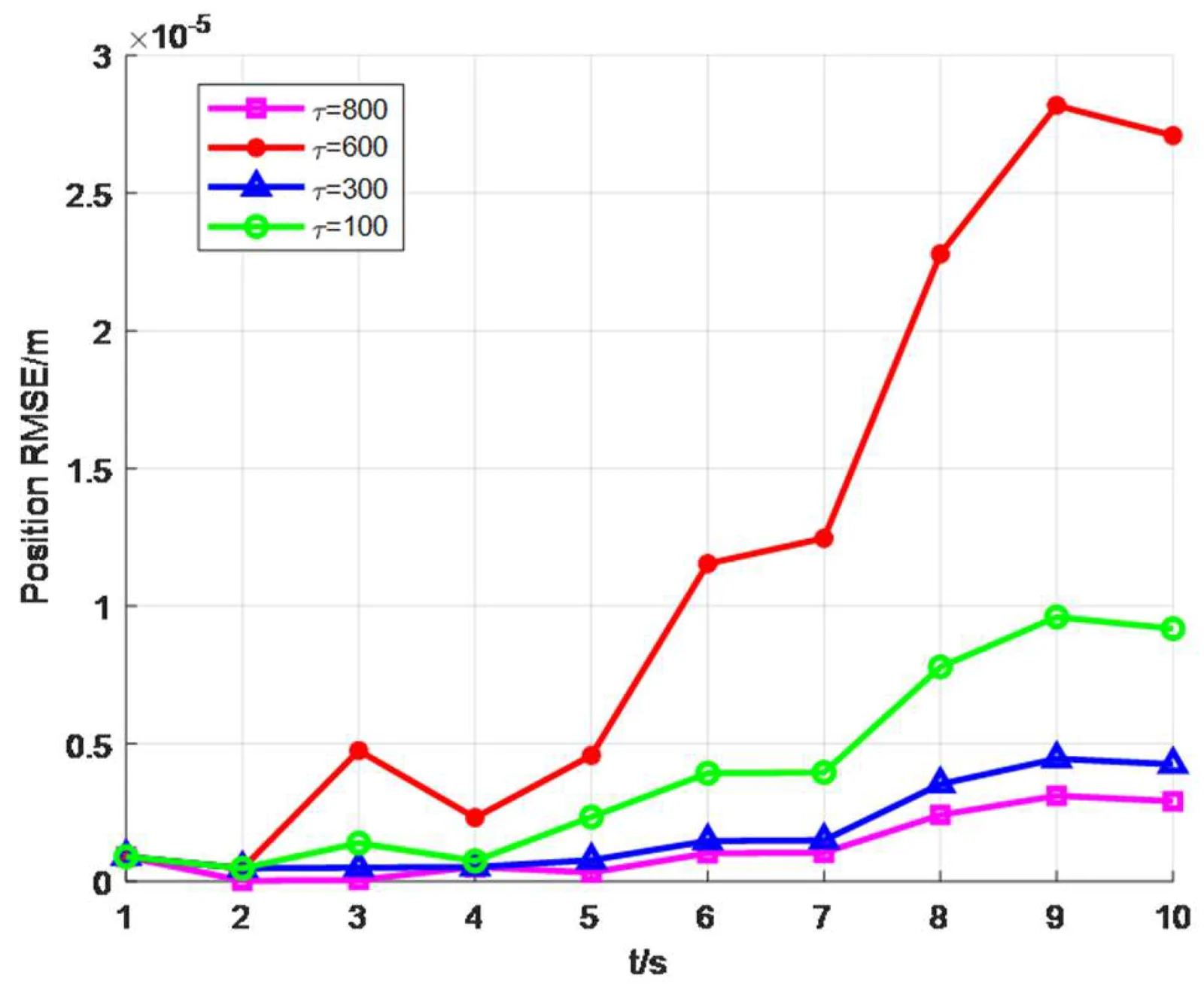
Position RMSE under different values of τ.

**Figure 8:**
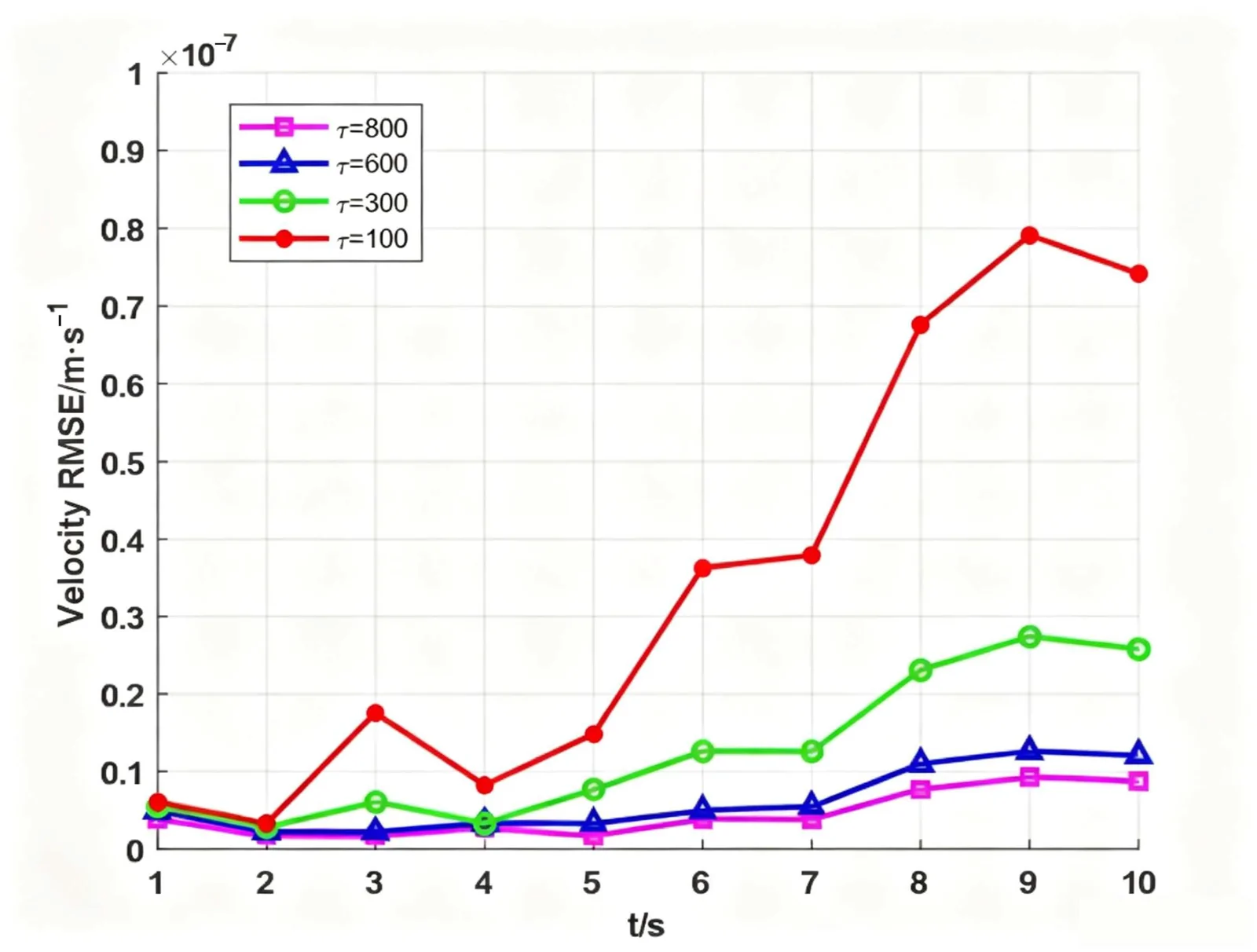
Velocity RMSE under different values of τ.

Figure 7 and Figure 8 respectively present the position and velocity RMSE of the proposed algorithm when τ=100, 300, 600, 800. As can be seen from Figure 7 and Figure 8, the proposed estimation method has a wide tuning range. Even with large variations in the value of τ, the position and velocity RMSE of the algorithm remain at low levels, demonstrating strong robustness.

**Figure 9:**
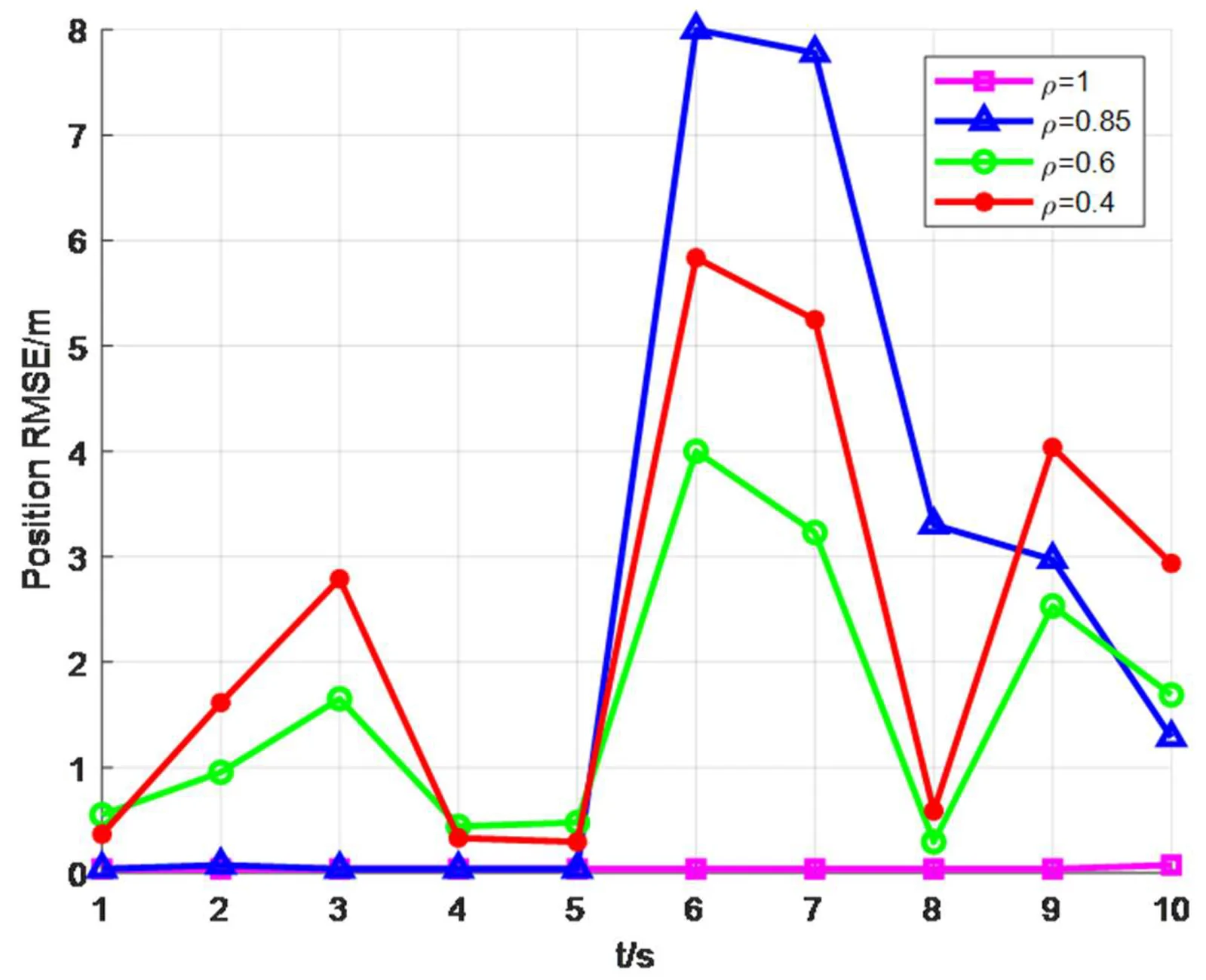
Position RMSE under different values of ρ.

**Figure 10:**
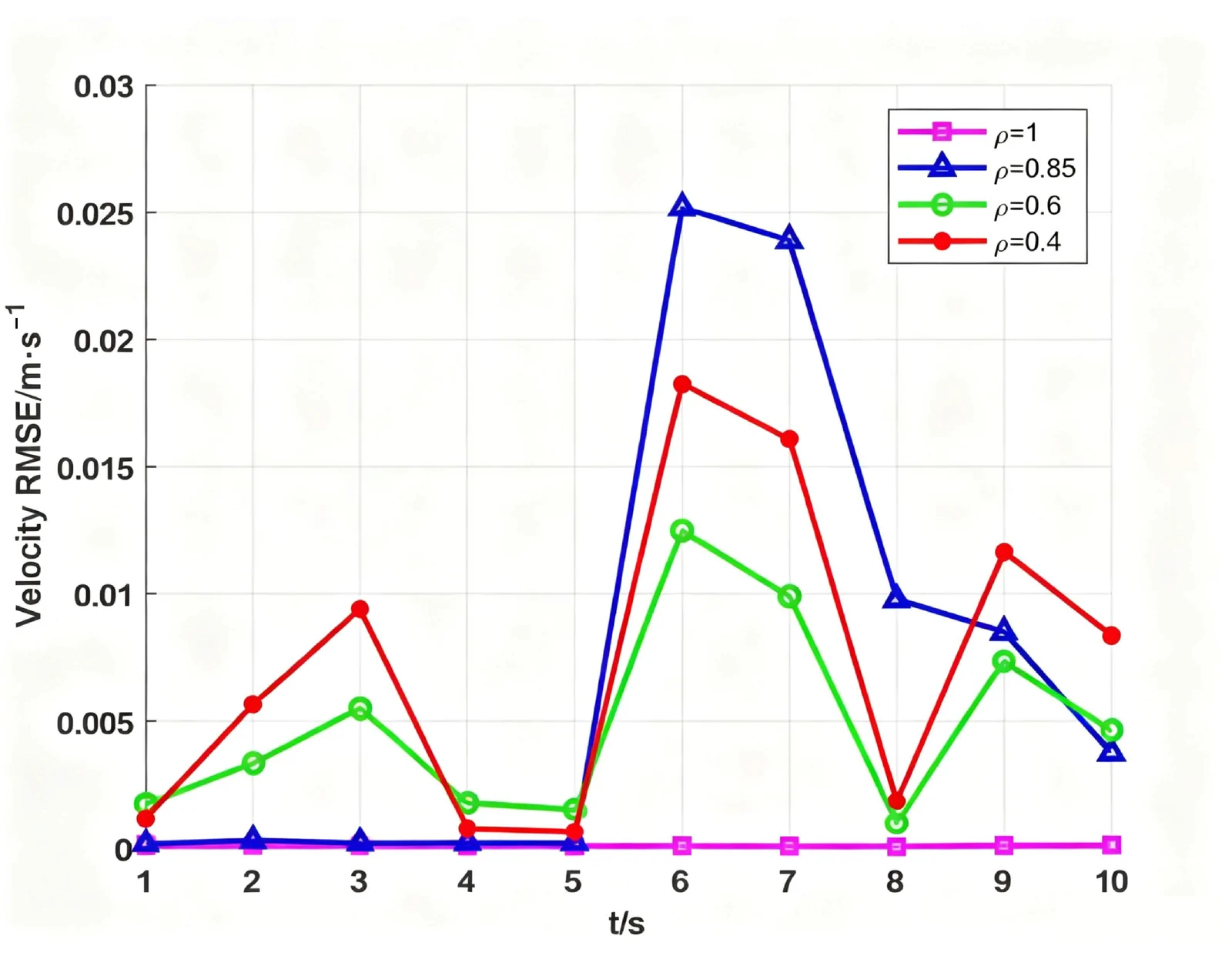
Velocity RMSE under different values of ρ.

Figure 9 and Figure 10 show the position and velocity RMSE results when ρ=0.4,0.6,0.85,1. It can be seen from the figures that the proposed algorithm has a wider selection range for ρ∈[0.4,  1] compared with existing algorithms, which are typically restricted to ρ∈[0.9,  1], while maintaining higher estimation accuracy than the existing methods.

## 4. Conclusions

In this paper, an adaptive estimation method for heterogeneous group targets considering uncertain multiplicative and additive noises is proposed, in which the multiplicative noise causes the marginal predictive measurement distribution to exhibit non-Gaussian heavy-tailed characteristics. A tailored hierarchical Gaussian–Gamma model is used to approximate the likelihood function. Within the Bayesian framework, the unknown process noise covariance and the unified measurement noise covariance are characterized as inverse Wishart priors, and through variational Bayesian fixed-point iteration, the dynamic states, extended shapes, and noise parameters are jointly estimated. Experimental results demonstrate that the proposed method can accurately estimate the motion states and shapes of heterogeneous group targets under multiplicative noise interference conditions.

## Figures and Tables

**Table 1:** Initial spatial configuration and measurement characteristics of the sub-targets.

Target Information	Position X	Position Y	Poisson Mean $\lambda_{i}$
Sub-Target 1	0 m	0 m	50
Sub-Target 2	−15 m	30 m	20
Sub-Target 3	15 m	30 m	20
Sub-Target 4	−15 m	−30 m	20
Sub-Target 5	15 m	30 m	20

**Table 2:** ARMSE comparison of evaluated algorithms.

Algorithms	Proposed Algorithm	RMM	VB-EOT-SN	Oracle RMM
Position ARMSE (m)	0.07 ± 0.01	315.95 ± 12.45	315.89 ± 12.38	0.35 ± 0.07
Velocity ARMSE (m/s)	0.08 ± 0.01	5.96 ± 0.23	5.95 ± 0.21	2.85 ± 0.15

**Table 3:** AMHD and AIoU comparison of evaluated algorithms.

Algorithms	Proposed Algorithm	RMM	VB-EOT-SN	Oracle RMM
AMHD (m)	26.01 ± 1.15	312.67 ± 15.30	311.78 ± 15.12	48.35 ± 3.42
AIoU	0.87 ± 0.02	0.03 ± 0.01	0.02 ± 0.01	0.45 ± 0.03

## Data Availability

The original contributions presented in this study are included in the article. Further inquiries can be directed to the corresponding author.
